# Supplementary material for: A yeast phenomic model for the influence of Warburg metabolism on genetic buffering of doxorubicin
Source: Cancer Metab. 2019 Oct 23;7:9. doi: 10.1186/s40170-019-0201-3 (PMC6806529; doi:10.1186/s40170-019-0201-3)
Supplement: Supplementary file 9 — Additional file 9. GO term-specific heatmaps for REMc/GTF-enriched clusters. GO term-specific heatmaps for significant GO process terms were generated as described in methods and Figs. 3 and 4. Any related child terms are presented in subsequent pages of the parent file name. GO terms with more than 100 children, with 2 or fewer genes annotated to the term, or a file size over 300KB are not shown. All heatmaps are generated with the same layout (see Figs. 3 and 4). [file 40170_2019_201_MOESM9_ESM.bz2 › Additional_File9_GOTermHeatmaps/Additional_File9_GOTermHeatmap/retrograde_transport,_endosome_to_Golgi.pdf]

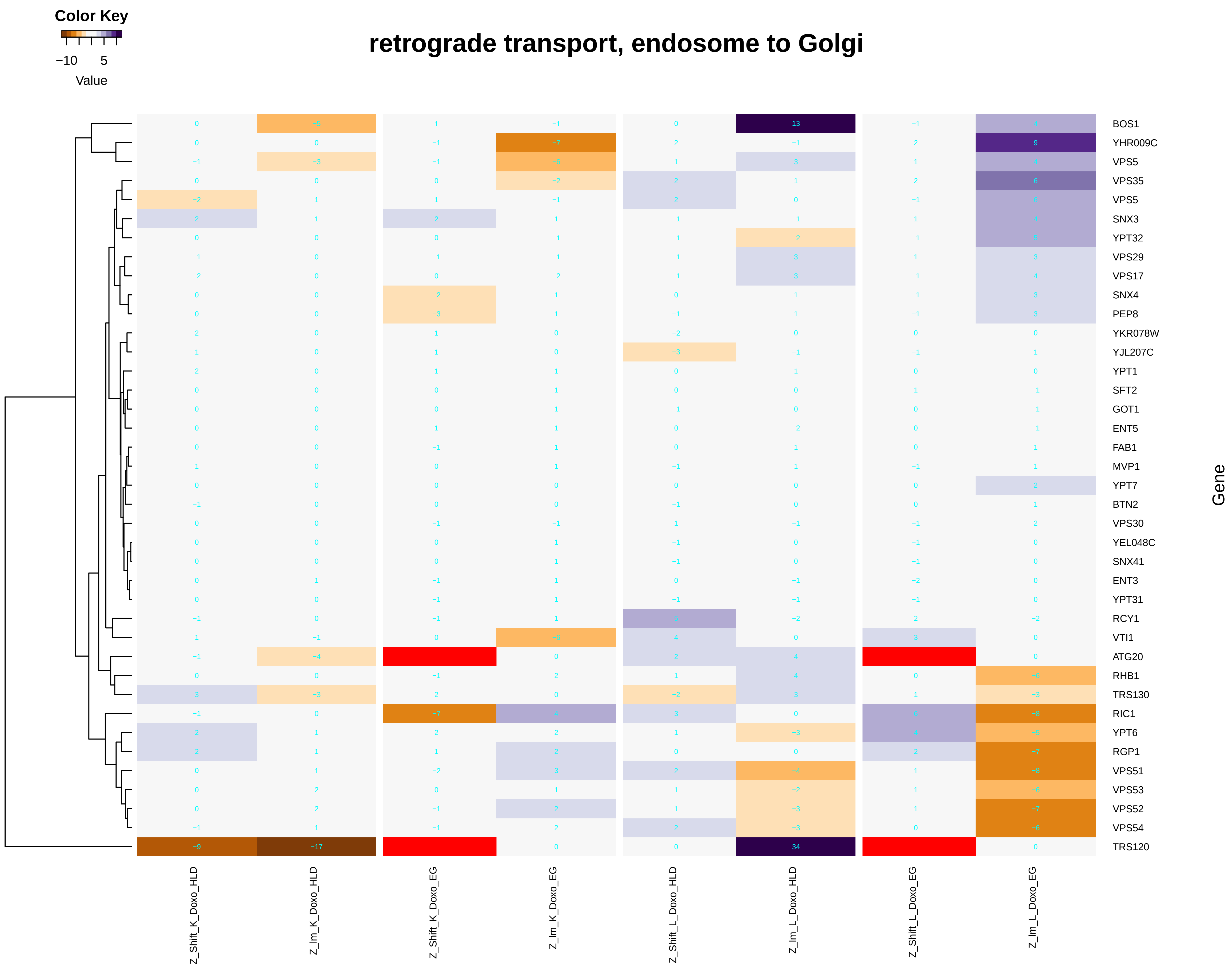
retrograde transport, endosome to Golgi
Color Key
−10
5
Value
Gene
BOS1
YHR009C
VPS5
VPS35
VPS5
SNX3
YPT32
VPS29
VPS17
SNX4
PEP8
YKR078W
YJL207C
YPT1
SFT2
GOT1
ENT5
FAB1
MVP1
YPT7
BTN2
VPS30
YEL048C
SNX41
ENT3
YPT31
RCY1
VTI1
ATG20
RHB1
TRS130
RIC1
YPT6
RGP1
VPS51
VPS53
VPS52
VPS54
TRS120
Z_Shift_K_Doxo_HLD
Z_Im_K_Doxo_HLD
Z_Shift_K_Doxo_EG
Z_Im_K_Doxo_EG
Z_Shift_L_Doxo_HLD
Z_Im_L_Doxo_HLD
Z_Shift_L_Doxo_EG
Z_Im_L_Doxo_EG

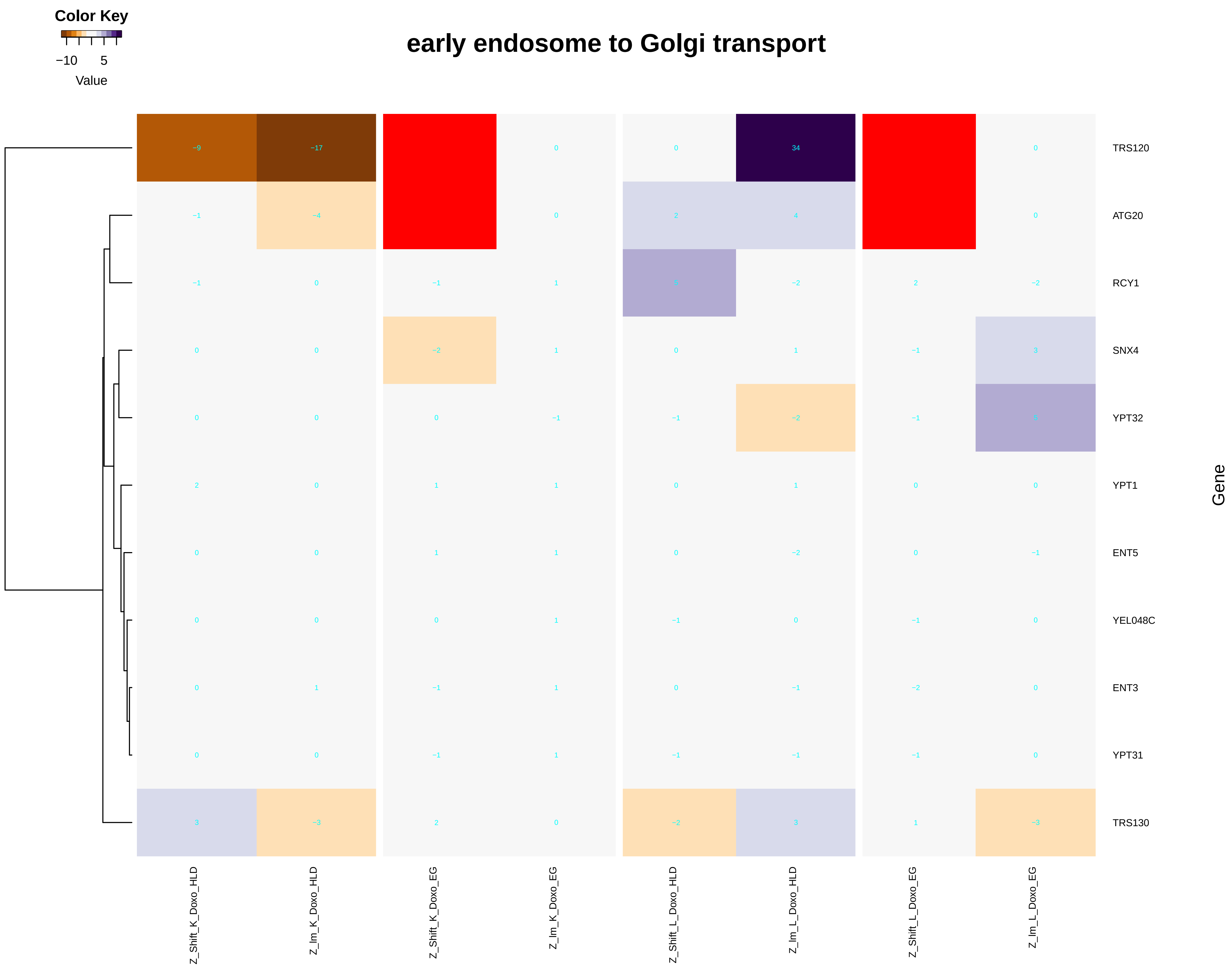

Color Key
−10
5
Value
early endosome to Golgi transport
TRS120
ATG20
RCY1
SNX4
YPT32
YPT1
ENT5
YEL048C
ENT3
YPT31
TRS130
Gene
Z_Shift_K_Doxo_HLD
Z_Im_K_Doxo_HLD
Z_Shift_K_Doxo_EG
Z_Im_K_Doxo_EG
Z_Shift_L_Doxo_HLD
Z_Im_L_Doxo_HLD
Z_Shift_L_Doxo_EG
Z_Im_L_Doxo_EG
